# Lack of association between circulating apelin level and frailty-related functional parameters in older adults: a cross-sectional study

**DOI:** 10.1186/s12877-020-01837-9

**Published:** 2020-10-21

**Authors:** Il-Young Jang, Seungjoo Lee, Jeoung Hee Kim, Eunju Lee, Jin Young Lee, So Jeong Park, Da Ae Kim, Mark W. Hamrick, Jin Hoon Park, Beom-Jun Kim

**Affiliations:** 1 Division of Geriatrics, Department of Internal Medicine, Asan Medical Center, University of Ulsan College of Medicine, Seoul, South Korea; 2 Department of Neurological Surgery, Asan Medical Center, University of Ulsan College of Medicine, 88 Olympic-ro 43-gil, Songpa-gu, Seoul, 05505 South Korea; 3 Asan Institute for Life Sciences, Asan Medical Center, University of Ulsan College of Medicine, Seoul, South Korea; 4 Department of Cell Biology and Anatomy, Medical College of Georgia, Augusta University, Augusta, GA USA; 5 Division of Endocrinology and Metabolism, Department of Internal Medicine, Asan Medical Center, University of Ulsan College of Medicine, 88 Olympic-ro 43-gil, Songpa-gu, Seoul, 05505 South Korea

**Keywords:** Apelin, Frailty, Aging, Sarcopenia, Biomarker

## Abstract

**Background:**

Apelin, an active endogenous peptide, has been recently receiving great attention as a promising target for antiaging intervention, primarily based on results from genetically altered mice. To validate previous experimental data and investigate the possible role of apelin in humans, in this study, we examined serum apelin level in relation to frailty and its associated parameters in a cohort of ambulatory, community-dwelling older adults.

**Methods:**

Blood samples were collected from 80 participants who underwent a comprehensive geriatric assessment, and apelin level was measured using an enzyme immunoassay kit. Phenotypic frailty and deficit-accumulation frailty index (FI) were assessed using widely validated approaches, proposed by Fried and Rockwood groups, respectively.

**Results:**

After adjustment for sex, age, and body mass index, serum apelin level was found to be not significantly different according to phenotypic frailty status (*P* = 0.550) and not associated with FI, grip strength, gait speed, time to complete 5 chair stands, and muscle mass (*P* = 0.433 to 0.982). To determine whether the association between serum apelin level and frailty has a threshold effect, we divided the participants into quartiles according to serum apelin level. However, there were no differences in terms of frailty-related parameters and the risk for frailty among the quartile groups (*P* = 0.248 to 0.741).

**Conclusions:**

The serum apelin level was not associated with both phenotypic frailty and functional parameters in older adults, despite its beneficial effects against age-related physiologic decline in animal models. Further large-scale longitudinal studies are necessary to understand the definite role of circulating apelin in frailty risk assessment.

**Supplementary information:**

**Supplementary information** accompanies this paper at 10.1186/s12877-020-01837-9.

## Background

Frailty is a geriatric condition characterized by progressive physiologic decline across multiple body systems, leading to increased vulnerability to adverse outcomes and death [1]. Because the number of older persons is rapidly increasing worldwide, frailty is likely to become a significantly more serious public health concern. Despite the lack of gold standard measurement, two major frailty models have been well accepted and widely used in aging research and practice [2]. The first concept proposed by Fried et al. [3] is “physical frailty” that views frailty as a biologic syndrome of decreased reserve and resistance to stressors. The other concept proposed by Rockwood et al. [4] is “cumulative deficit frailty,” which is also termed as “frailty index (FI)”, based on the hypothesis that the accumulation of health, functional, psychological, and cognitive problems serves as an indicator of an individual’s aging-related health status. Although each assessment tool has strengths and weaknesses in terms of clinical application [2], both are useful to identify older adults at high risk for mortality [5]. Importantly, frailty is a heterogeneous condition resulting from various causes that might be potentially reversible [6]. Therefore, continuous efforts to identify factors affecting functional changes with aging are critical to achieve the goal of expanding health span.

Apelin is an active endogenous peptide that acts via G protein-coupled apelin receptor (APJ) to exert diverse functional effects [7]. This pleiotropic molecule is involved in multiple biologic processes, including cardiovascular regulation, neuroprotection, immunity, and glucose metabolism [8–10]. Apelin has been recently receiving great attention as a promising target for antiaging intervention, primarily based on results from genetically altered mice [11]. For example, the expression of apelin and APJ was downregulated in an age-dependent manner [12, 13]. Importantly, apelin-deficient mice exhibited accelerated the onset of sarcopenia and senescence, whereas apelin treatment in aged mice enhanced muscle strength and physical activity and rejuvenated behavioral and circadian phenotypes [12, 13]. However, despite the clear implications of apelin in physiologic alterations with aging, clinical studies relating circulating apelin concentration to frailty are limited. With the aim to validate previous in vitro and in vivo data and investigate the possible role of apelin in humans, this study examined serum apelin level in relation to frailty and its associated parameters in a cohort of older adults.

## Methods

### Study design, setting, and participants

This was a cross-sectional outpatient-based study. The study population comprised Koreans who had undergone a comprehensive geriatric assessment (CGA) at the Division of Geriatrics, Department of Internal Medicine, Asan Medical Center (AMC, Seoul, Korea), between July 2019 and April 2020. These participants had visited the clinic for the management of chronic diseases, such as hypertension, hyperlipidemia, and osteoarthritis, or for the evaluation of non-specific symptoms, such as fatigue and loss of appetite, which are frequently observed in older adults. They were ambulatory, community-dwelling, and not from nursing homes or inpatient facilities. Participants whose life expectancy was expected to be less than 1 year due to malignancy, symptomatic heart failure, or end-stage renal failure were excluded. After the exclusion of ineligible participants, we collected blood samples from 80 eligible participants at the same visit when CGA was performed with their consent.

### Ethics statement

This study was approved by the AMC Institutional Review Board (no. 2020–0259). Written informed consent was obtained from all enrolled participants. Ethical measures were in concordance with the Declaration of Helsinki.

### Comprehensive geriatric assessment

All participants underwent a CGA by experienced nurses. Information on demographic characteristics and medical or surgical histories was collected through detailed interviews and reviews of medical records. We used measures from previously validated CGA-frailty index (CGA-FI) variables [14], including the geriatric domains of comorbidities; functional status; physical performance; nutritional status; and common geriatric syndromes, such as cognitive dysfunction, depression, or polypharmacy.

Multimorbidity was defined as having two or more of the 18 physician-diagnosed illnesses, namely, angina, atrial fibrillation/flutter, coronary artery disease, diabetes, heart failure, hypertension, myocardial infarction, peripheral vascular disease, stroke, anxiety disorder, arthritis, asthma, cancer within 5 years, chronic kidney disease (estimated glomerular filtration rate < 60), chronic obstructive lung disease, degenerative spine disease, depression, and sensory impairment. Disability was defined as requiring assistance from another person to perform any of the seven activities of daily living (ADLs; feeding, dressing, grooming, walking, getting in and out of bed, toileting, and bathing or showering) or seven instrumental ADLs (IADLs) (making telephone calls, using transportation, shopping, cooking, doing housework, taking medications, and managing money). To assess social frailty, the 5-item social frailty questionnaire was administered: (1) going out less frequently, (2) rarely visiting the homes of friends, (3) feeling unhelpful to friends and family, (4) being alone, and (5) not talking with someone every day [15]. Cognitive dysfunction was defined as having less than 24 points on mini-mental status examination on selective participants identified as positive in the mini-cognitive screening test [16]. Depression was considered when the score of the 15-item Korean version of the short form of geriatric depression scale was 10 or more on selective participants identified as positive in the patient health questionnaire-2 (PHQ-2) screening test [17].

### Functional status and sarcopenia assessment

Handgrip strength of the dominant side was measured using Jamar hydraulic hand dynamometer (Patterson Medical, Warrenville, IL, USA) [18]. Participants were instructed to sit comfortably, bend their elbow at 90 degrees, and hold the dynamometer as strong as possible. The maximum value was adapted after all tests were conducted twice at intervals of 1 min or more. Usual gait speed (meters per second) from a 4-m walk and time to complete 5 chair stands (seconds) were measured [19]. The short physical performance battery (SPPB) comprises repeated chair stands, standing balance, and gait speed [20]. In the standing balance test, including side-by-side stance, semi-tandem stance, and tandem stance, the participants were instructed to stand for up to 10 s. Ranging from 0 to 12 points, a higher SPPB score means better lower extremity function.

Body compositions including muscle mass (whole body lean body mass minus bone mineral content) were evaluated using a bioelectrical impedance analyzer (InBody S10; InBody, Seoul, Korea) with measuring frequencies of 1, 5, 50, 250, 500, and 1000 kHz [21]. Participants were instructed to fast for > 8 h before the examination to minimize the possible effects of food and water intake. Appendicular skeletal muscle mass (ASM) was calculated as the sum of the muscle mass of both arms and legs, and ASM index (ASMI) was defined as adjusting the ASM to the height squared for an objective comparison of muscle mass between participants [22]. Finally, sarcopenia was diagnosed using the 2019 Consensus Guidelines from the Asian Working Group for Sarcopenia [23]. Briefly, older patients with low muscle mass (ASMI < 7.0 kg/m^2^ in men, < 5.7 kg/m^2^ in women) and low muscle strength (handgrip strength < 28 kg in men, < 18 kg in women) and/or low physical performance (gait speed < 1.0 m/s, 5-time chair stand test ≥12 s, or SPPB score ≤ 9 points) were classified as having sarcopenia.

### Frailty assessment

1) Phenotypic frailty: Frailty was evaluated according to the Cardiovascular Health Study frailty criteria, a widely validated definition for frailty, proposed by Fried et al. [3]. The frailty phenotype scale was calculated by assigning 1 point to each of the five components that are relevant to an individual [24]: self-reported exhaustion determined by PHQ-2 with a total score > 2, low physical activity defined as weekly activity amount < 494.65 kcal for men and < 283.50 kcal for women using the International Physical Activity Questionnaire (IPAQ), weakness determined by the grip strength of the lowest quintile corresponding to sex and body mass index (BMI), slowness determined by the gait speed of the lowest quintile corresponding to sex and height, and unintentional weight loss > 4.5 kg in the past 12 months. How these assessments were performed in our study has been previously described [24]. Based on the total score, individuals were classified as robust (0 points), prefrail (1–2 points), or frail (3–5 points).

2) Deficit-accumulation frailty index (FI): The FI is known as the most sensitive predictor of adverse health outcomes and is based on the cumulative effect of medical, functional, and psychosocial age-related deficits, proposed by Rockwood et al. [4]. In this study, we calculated a FI that has been validated in other studies (see the complete list of assessed items in Supplementary Material) [2, 14]. The ratio between the number of identified deficits and 50 evaluable items is calculated from 0 to 1, indicating that the higher the FI, the greater the frailty status.

### Measurement of apelin in human serum

Blood samples were collected from the antecubital vein of each participant in the morning after an overnight fast of at least 8 h. After sample centrifugation at 3000 rpm for 5 min at 4 °C, we carefully collected the supernatants to exclude cell components. All samples with hemolysis or clotting were discarded. The serum samples were stored at − 80 °C prior to the determination of concentrations. Serum apelin concentration was measured using a previously validated nonselective apelin-12 enzyme immunoassay kit that recognizes the C-terminal sequence of 12 amino acids shared among all apelin isoforms (Phoenix Pharmaceuticals, Belmont, CA, USA) [13, 25]. The lower limit of detection of the kit was 0.07 ng/mL, and the intra- and inter-assay coefficients of variation were less than 10 and 15%, respectively.

### Sample size estimation

In this study, we determined the sample size in accordance with the observations from previous human studies. Because of the limited clinical data on serum apelin levels according to the frailty spectrum, we used previously available data on serum apelin levels in diabetic patients [mean, 0.68; standard deviation (SD), 0.40; *n* = 228] and non-diabetic patients (mean, 0.44; SD, 0.29; *n* = 162) [26]. Although direct comparison between diabetes and frailty might be inappropriate, we performed this comparison considering that diabetes is one of the known phenotypes associated with metabolic aging. The required sample size for each group, with an alpha of 0.05 and a beta of 0.80, was 23 if the frailty spectrum was classified into two groups. Similarly, a study measuring serum apelin levels in non-sarcopenic and sarcopenic patients showed significant between-group differences in apelin levels in 61 people [13]; however, these data could not be used in power calculations because of the lack of information on SD. As the frailty spectrum is continuous in terms of the FI and frailty is commonly classified into three groups according to frailty phenotype, we predetermined that a study population of 80 would be sufficient to assess serum apelin levels according to frailty status.

### Statistical analyses

All data are presented as means ± standard deviation or as numbers and percentages, unless otherwise specified. Baseline characteristics of the study participants according to phenotypic frailty status were compared using the analysis of variance with post-hoc analysis via Tukey’s honest significance test for continuous variables and Fisher’s exact tests for categorical variables. The estimated means with 95% confidence intervals in serum apelin level according to the phenotypic frailty status and in frailty-related parameters according to serum apelin quartiles were generated and compared using analysis of covariance before and after adjustment for sex, age, and body mass index (BMI). The association between serum apelin level and frailty-related parameters was investigated using the linear regression analysis. To generate the odds ratio (OR) for phenotypic frailty according to serum apelin quartiles, logistic regression analysis was performed. All statistical analyses were performed using the Statistical Package for the Social Sciences (SPSS) version 18.0 (SPSS Inc., Chicago, IL, USA). *P* < 0.05 was considered statistically significant.

## Results

Baseline characteristics of 80 study participants are listed in Table 1. Among the 21 (26.2%) robust, 47 (58.8%) prefrail, and 12 (15.0%) frail older adults based on Fried’s criteria, 8 (38.1%), 28 (59.6%), and 7 (58.3%) were women, respectively. The mean ages of the robust, prefrail, and frail groups were 67.5 ± 6.5, 69.7 ± 6.2, and 70.8 ± 5.0, respectively. There were no significant differences in weight, height, BMI, serum albumin level, time to complete 5 chair stands, ASMI, and prevalence of polypharmacy, multimorbidity, and ADL disability among the three groups. Frail participants had lower grip strength, gait speed, SPPB score, muscle mass, and mini-cognitive score and higher FI, social frailty score, and PHQ-2 score than robust and/or prefrail participants. Furthermore, frail participants were more likely to have sarcopenia, IADL disability, cognitive dysfunction, and depression than robust and/or prefrail participants.

**Table 1:** Baseline characteristics of the study participants according to phenotypic frailty status

Variables	Robust (*n* = 21)	Prefrail (*n* = 47)	Frail (*n* = 12)	*P*
Sex, no. (%)				0.245
Female	8 (38.1)	28 (59.6)	7 (58.3)	
Male	13 (61.9)	19 (40.4)	5 (41.7)	
Age (years)	67.5 ± 6.5	69.7 ± 6.2	70.8 ± 5.0	0.257
Weight (kg)	71.7 ± 11.3	67.3 ± 10.2	63.3 ± 8.6	0.069
Height (cm)	161.7 ± 10.0	158.9 ± 8.7	157.2 ± 5.6	0.313
Body mass index (kg/m^2^)	27.4 ± 3.2	26.6 ± 3.0	25.7 ± 3.8	0.297
Serum albumin (g/dL)	3.83 ± 0.36	3.84 ± 0.28	3.73 ± 0.25	0.559
Frailty index (range, 0–1)	**0.070 ± 0.035**	0.126 ± 0.063^*^	0.230 ± 0.115^*,†^	**< 0.001**
Grip strength (kg)	**32.7 ± 7.8**	**27.9 ± 8.9**	20.9 ± 6.6^*,†^	**0.001**
Gait speed (m/s)	**1.21 ± 0.18**	**1.03 ± 0.33**	0.75 ± 0.28^*,†^	**< 0.001**
Chair stand (s)	9.6 ± 4.2	11.3 ± 8.2	14.5 ± 7.2	0.193
SPPB score (range, 0–12)	**11.5 ± 1.0**	**10.3 ± 2.7**	9.5 ± 2.2^*^	**0.046**
Muscle mass (kg)	**25.3 ± 4.9**	**22.5 ± 5.6**	20.0 ± 3.6^*^	**0.021**
ASMI (kg/m^2^)	7.29 ± 1.26	6.78 ± 1.26	6.23 ± 0.99	0.065
Sarcopenia, no. (%)	**0 (0.0)**	**7 (14.9)**	**5 (41.7)**	**0.006**
Use of ≥5 prescription drugs, no. (%)	8 (38.1%)	23 (48.9)	8 (66.7)	0.287
Multimorbidity, no. (%)	15 (71.4)	35 (74.5)	11 (91.7)	0.382
ADL disability, no. (%)	0 (0.0)	3 (6.4)	2 (16.7)	0.163
IADL disability, no. (%)	**1 (4.8)**	**15 (31.9)**	**8 (66.7)**	**0.001**
Social frailty score (range, 0–5)	**0.95 ± 0.67**	**1.40 ± 1.10**	2.08 ± 1.00^*^	**0.009**
Mini-Cognition score (range, 0–5)	**4.33 ± 0.86**	**3.64 ± 1.24**	3.27 ± 1.27^*^	**0.026**
Cognitive dysfunction, no. (%)	**0 (0.0)**	**3 (6.4)**	**4 (33.3)**	**0.003**
PHQ-2 score (range, 0–6)	**0.86 ± 0.85**	**1.45 ± 1.94**	3.82 ± 1.72^*,†^	**< 0.001**
Depression, no. (%)	**0 (0.0)**	**5 (10.6)**	**8 (66.7)**	**< 0.001**

Values are presented as the mean ± standard deviation unless otherwise specified. **Bold** means that values are statistically significant. Comparisons between the three groups were investigated using the analysis of variance with post-hoc analysis via Tukey’s honest significance test for continuous variables and Fisher’s exact tests for categorical variables. * and † indicate statistically significant differences of continuous variables from the robust and prefrail groups, respectively. *SPPB* short physical performance battery, *ASMI* appendicular skeletal muscle mass index, *ADL* activity of daily living, *IADL* instrumental activity of daily living, *PHQ-2* patient health questionnaire-2

**Fig. 1:**
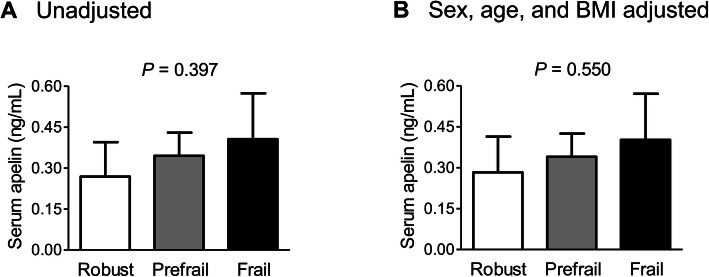
Differences in serum apelin levels according to the phenotypic frailty status (**a**) before and (**b**) after adjustment for sex, age, and BMI. The estimated means with 95% confidence intervals were generated and compared using analysis of covariance. BMI, body mass index

Differences in serum apelin level according to the phenotypic frailty status were assessed using analysis of covariance. Before and after adjustment for sex, age, and BMI, there were no significant differences in serum apelin level between the three groups (Fig. 1).

Linear regression analyses were performed to examine the association between serum apelin level and frailty-related parameters with normal distribution (Table 2). However, serum apelin concentration was not associated with FI, grip strength, gait speed, time to complete 5 chair stands, muscle mass, and ASMI, regardless of the adjustment models.

**Table 2:** Linear regression analyses to determine the association between serum apelin level and frailty-related parameters

	Unadjusted				Sex, age, and BMI adjusted			
	β	SE	*β*	*P*	β	SE	*β*	*P*
Frailty index	0.022	0.032	0.076	0.503	0.017	0.031	0.058	0.598
Grip strength	− 4.825	3.462	− 0.157	0.167	− 0.558	2.183	− 0.018	0.799
Gait speed	−0.153	0.123	−0.142	0.216	−0.093	0.118	−0.086	0.433
Chair stand	−1.181	2.842	−0.048	0.679	−2.134	2.811	−0.086	0.450
Muscle mass	−3.102	2.073	−0.168	0.139	0.025	1.081	0.001	0.982
ASMI	−0.645	0.486	−0.150	0.189	0.051	0.330	0.012	0.876

The Enter method was applied to this model. Bold means that values are statistically significant. β unstandardized regression coefficient, *SE* standard error, *β* standardized regression coefficient, *BMI* body mass index, *ASMI* appendicular skeletal muscle mass index

**Fig. 2:**
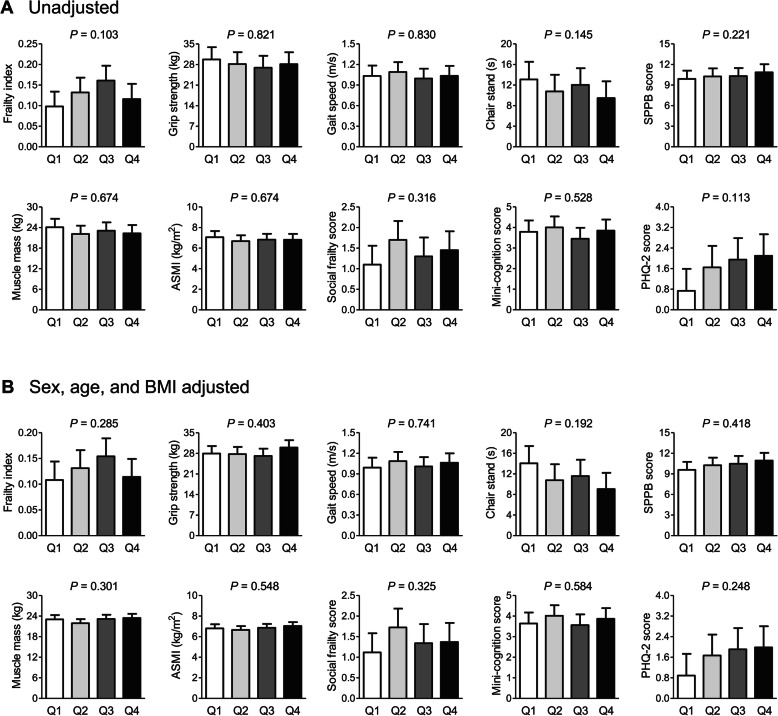
Differences in frailty-related parameters according to serum apelin quartiles (**a**) before and (**b**) after adjustment for sex, age, and BMI. The estimated means with 95% confidence intervals were generated and compared using analysis of covariance. Serum apelin quartiles: Q1 = 0.087–0.199 ng/mL, Q2 = 0.200–0.269 ng/mL, Q3 = 0.270–0.378 ng/mL, and Q4 = 0.379–2.440 ng/mL. BMI, body mass index; SPPB, short physical performance battery; ASMI, appendicular skeletal muscle mass index; PHQ-2, patient health questionnaire-2

**Fig. 3:**
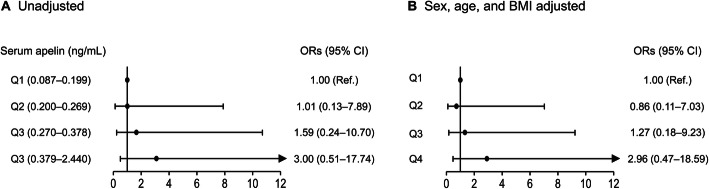
Logistic regression analyses to determine the odds ratios for phenotypic frailty according to serum apelin quartiles (**a**) before and (**b**) after adjustment for sex, age, and BMI. BMI, body mass index; OR, odds ratio; CI, confidence interval

To determine whether the association between serum apelin level and frailty-related parameters may have a threshold effect rather than a gradual effect, we divided the participants into quartiles according to serum apelin level (Fig. 2). Before and after adjustment for sex, age, and BMI, the groups did not differ significantly in terms of FI, grip strength, gait speed, time to complete 5 chair stands, SPPB score, muscle mass, ASMI, social frailty score, mini-cognitive score, and PHQ-2 score. Furthermore, logistic regression analyses showed that the ORs for phenotypic frailty according to serum apelin quartiles were not statistically significant, regardless of the adjustment models (Fig. 3).

## Discussion

In a cohort of ambulatory, community-dwelling older adults, we observed that serum apelin level was not significantly associated with both the phenotypic frailty and the functional parameters. Additionally, serum apelin level was not associated with the risk of phenotypic frailty before and after adjustment for sex, age, and BMI.

Many lines of evidence from in vitro and animal experiments indicate that apelin may be implicated in various age-related disorders. While mice lacking apelin and its receptor showed markedly decreased muscle strength and physical performance, chronic apelin supplementation attenuated muscle aging [8, 13]. The beneficial effects of apelin on muscle metabolism are probably mediated by the stimulation of AMP-activated protein kinase and Akt and the resultant mitochondriogenesis [13, 27]. Downregulation of apelin in mice exhibited age-dependent decline in multiple organs, whereas systemic restoration of apelin improved cognitive and cardiovascular dysfunction, resulting in increased mammalian health span [8, 9, 11, 12, 28]. Because these physiologic alterations including musculoskeletal weakness and neurodegeneration are major manifestations of frailty, apelin could be expected not only as a therapeutic target to slow or reverse aging process but also as a clinical biomarker to assess the frailty status.

Despite the definite role and plausible mechanisms of apelin as an antiaging factor in animal models, its effects on human health remain unclear. Because it is impossible to conduct interventional trials in humans without safety verification from preclinical data, the roles of candidate factors on human metabolism must be extrapolated from the observational results, leading us to conduct this clinical study. However, different from the experimental results, we did not observe a significant association between circulating apelin level and frailty-related parameters in older adults. Although we cannot determine the exact reason for this discrepancy, several explanations might be hypothesized. First, although mice are widely used to study human illness mechanisms because of biologic similarity, they are not the same. In detail, the comparison research focusing on the genetic and biochemical processes regulating genome activity in humans and mice by the Mouse ENCODE Consortium revealed a large degree of divergence of sequences involved in transcriptional regulation, chromatin state, and higher-order chromatin organization [29, 30]. This could answer the question why certain processes and systems in mice, such as the immune system, metabolism, and stress response, are significantly different to those in humans and why drugs that have worked in animal experiments are not equally successful in humans. The lack of associations between apelin and frailty in humans, unlike in mice, could also be attributable to these differences in two mammals. Second, physiologic apelin level observed in humans may not be high enough to exert the phenotypic changes. The apelin expression and production are almost completely blocked in apelin-deficient mice, and the concentration of apelin treated into mice is relatively high with a long period [12, 13]. On the contrary, because the difference in apelin mean values between the lowest (Q1) and highest (Q4) quartiles in our study was only 0.466 ng/mL, we may have not observed significant changes of functional parameters. Third, apelin is synthesized as a 77-residue preproapelin and cleaved into the 55-residue proapelin and subsequently into 13–36-residue active isoforms [31]. Therefore, diverse isoforms, such as apelin-13, apelin-17, and apelin-36, could be present in biological fluids, including blood, and there is a possibility that each isoform plays a different role as a biomarker to assess frailty. This issue needs to be clarified through further studies using more accurate techniques.

The major strengths of our study are that we adapted both frailty operational definitions that have been well validated and included various functional parameters, including grip strength, gait speed, time to complete 5 chair stands, SPPB score, social frailty score, mini-cognitive score, PHQ-2 score, as well as muscle mass, contributing to increased reliability of our results. However, despite these strengths, several limitations should also be considered when interpreting the results. First, because this was a cross-sectional study, we could not determine a causal relationship between the variables. Second, because of difficulties in simultaneously obtaining the information about frailty status and the blood samples, the number of enrolled participants with consent was relatively small. However, we believe that this preliminary analysis might provide an important background for further studies. Third, our study population was exclusively Koreans. Thus, we cannot be certain that our results are applicable to other populations. Fourth, we could not adjust for various confounding factors because of a relatively small sample size in the analysis. Finally, we cannot exclude the possibility that any biased information or uncontrolled factors that affect apelin and frailty could affect the conclusion.

## Conclusion

In summary, serum apelin levels did not differ significantly according to the phenotypic frailty status and was not associated with FI and functional parameters in older adults, despite the beneficial effects of apelin against age-related physiologic decline that is observed primarily in animal models. Additional well-designed large-scale longitudinal studies are necessary to understand the definite role of circulating apelin in frailty risk assessment.

## Supplementary information

**Additional file 1.**

## Data Availability

The datasets used and/or analysed during the current study are available from the corresponding author on reasonable request.

## Abbreviations

FI: Frailty index
APJ: G protein-coupled apelin receptor
CGA: Comprehensive geriatric assessment
AMC: Asan Medical Center
ADL: Activity of daily living
IADL: Instrumental activity of daily living
PHQ-2: Patient health questionnaire-2
SPPB: Short physical performance battery
ASM: Appendicular skeletal muscle mass
ASMI: Appendicular skeletal muscle mass index
BMI: Body mass index
OR: Odds ratio
