# Supplementary material for: Lack of association between circulating apelin level and frailty-related functional parameters in older adults: a cross-sectional study
Source: BMC Geriatr. 2020 Oct 21;20:420. doi: 10.1186/s12877-020-01837-9 (PMC7579806; doi:10.1186/s12877-020-01837-9)
Supplement: Supplementary file 1 — Additional file 1. [file 12877_2020_1837_MOESM1_ESM.docx]

**Supplementary Materials**

**Lack of Association between Circulating Apelin Level and Frailty-Related Functional Parameters in Older Adults**

Il-Young Jang, Seungjoo Lee, Jeoung Hee Kim, Eunju Lee, Jin Young Lee, So Jeong Park,

Da Ae Kim, Mark W. Hamrick, Jin Hoon Park, Beom-Jun Kim

**TABLE OF CONTENTS**

| **Supplementary Table.** | Items for comprehensive geriatric assessment-frailty index |
| --- | --- |
|  |  |

**Supplementary Table.** Items for comprehensive geriatric assessment-frailty index

| **Medical History (21 items)** | | | |
| --- | --- | --- | --- |
| • Angina  • Anxiety disorder  • Arthritis  • Asthma  • Atrial fibrillation/flutter  • Cancer within 5 years  • Chronic kidney disease (eGFR < 60) | • COPD  • Coronary artery disease  • Degenerative spine disease  • Dementia  • Depression  • Diabetes  • Fall within the past year | | • Heart failure  • Hypertension  • Myocardial infarction  • Peripheral vascular disease  • Sensory impairment  • Stroke/TIA  • Use of ≥ 5 prescription drugs |
| **Functional Status (22 items)** | | | |
| **Activities of Daily Living**  • Feeding  • Dressing/undressing  • Grooming  • Walking (or use of a walker)  • Getting in and out of bed  • Toileting  • Bathing or shower | **Activities of Daily Living**  • Using telephone  • Using transportation  • Shopping  • Preparing own meals  • Housework  • Taking own medications  • Managing money | | **Nagi and Rosow-Breslau Activities**  • Pulling or pushing a large object  • Stooping, crouching or kneeling  • Lifting or carrying 10 lbs  • Reaching arms above shoulder  • Writing or handling small objects  • Walking up/down a flight of stairs  • Heavy work around house |
| **Performance Tests (4 items)** | | | |
| **Mini-Mental Status Examination**  27–30 points (0 points)  24–26 points (0.3 points)  21–23 points (0.7 points)  <21 points (1 point) | | **5 Repeated Chair Stands**  <11.20 s (0 points)  11.20–13.69 s (0.25 points)  13.70–16.69 s (0.5 points)  16.70–60.9 s (0.75 points)  ≥ 61.0 s (1 point) | |
| **Gait Speed**  ≥ 1 m/sec (0 points)  0.80–0.99 m/s (0.3 points)  0.60–0.79 m/sec (0.7 points)  <0.60 m/s (1 point) | | **Dominant Handgrip Strength**  M, ≥ 32 kg; F, ≥ 20 kg (0 points)  M, ≥ 26–31 kg; F, 16–19 kg (0.5 points)  M, <26 kg; F, <16 kg (1 point) | |
| **Nutritional Status (3 items)** | |  | |
| • Weight loss > 4.5 kg in past year | • Body mass index < 21 kg/m^2^ | | • Serum albumin < 3.5 g/dL |

Abbreviations: COPD, chronic obstructive pulmonary disease; eGFR, estimated glomerular filtration rate; F, female; M, male; TIA, transient ischemic attack.
